# Sex‐Specific Aging Patterns of Gut Microbiota in Urban Chinese Adults: Guild‐Based Analysis and Implications for Healthy Aging

**DOI:** 10.1111/acel.70192

**Published:** 2025-08-04

**Authors:** Jiongxing Fu, Wanghong Xu, Danxia Yu, Yu Jiang, Lei Wang, Hui Cai, Qinghua Xia, Xiao‐Ou Shu, Wei Zheng

**Affiliations:** ^1^ Department of Epidemiology, NHC Key Laboratory for Health Technology Assessment Fudan University School of Public Health Shanghai China; ^2^ Division of Epidemiology, Department of Medicine Vanderbilt University Medical Center Nashville Tennessee USA; ^3^ Changning District Center for Disease Control and Prevention (Health Supervision Institute) Shanghai China

**Keywords:** aging, chronic disease, gastrointestinal microbiome, healthy lifestyle, sex characteristics

## Abstract

Gut microbial stability typically decreases with physiological aging. This decline may vary between sexes and can potentially be mitigated by adopting a healthy lifestyle. Microbial guilds, defined as functionally coherent groups of bacteria, may serve as meaningful ecological indicators of aging. This study included 2944 participants aged 51–89 years from the Shanghai Men's and Women's Health Studies. Using 16S rRNA gene sequencing and a guild‐based approach, we evaluated the associations between gut microbiota and age in 1353 relatively healthy individuals. We found that women demonstrated a decline in the Chao1 index, an increase in Pielou evenness, and a remarkable shift in Bray–Curtis distance, whereas men exhibited an increase in Bray–Curtis uniqueness. Of the 45 age‐related guilds identified, 16 (8 in men and 10 in women) were considered potential aging biomarkers (*p*_FDR_ < 0.05), with Guild_6 (*Bifidobacterium* sp. dominated) and Guild_118 ( *Veillonella dispar* dominated) being common to both sexes. These guilds were associated with consistent predicted functions, particularly 1,4‐dihydroxy‐2‐naphthoate biosynthesis. We estimated sex‐specific microbial age using random forest models and found that women and individuals with major chronic diseases had higher microbial ages. Prospective analysis revealed that an “old” microbial age was associated with a higher risk of type 2 diabetes (HR = 2.0, 95% CI: 1.1, 3.7). Individuals with healthier lifestyles had a 0.43‐year lower microbial age (95% CI: −0.85, −0.01). Our findings elucidate the sex‐differentiated aging patterns of gut microbiota in Chinese adults and imply the potential benefits of healthy lifestyle behaviors in slowing down microbiome aging.

## Graphical abstract

This study employed a guild‐based approach to identify sex‐specific age‐related gut microbiota in two large cohorts of Chinese adults. The microbial age, derived from the significant guilds identified, was higher in women and less healthy individuals, was associated with an increased risk of type 2 diabetes, and could be slowed by healthy lifestyles. These findings highlight the role of multiple major noncommunicable diseases in accelerating aging and underscore the importance of a healthy lifestyle in promoting healthy aging.

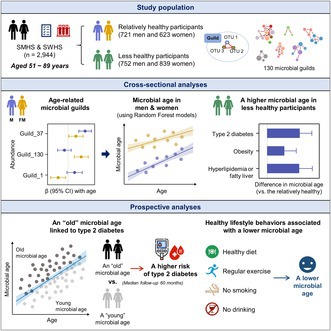

## 1 Introduction

Aging is a progressive decline in physiological integrity due to the interplay of genetic, environmental, epigenetic, and stochastic factors (López‐Otín et al. 2023). Characterized by increased vulnerability to external hazards, aging is a primary risk factor for multiple noncommunicable diseases (NCDs), including type 2 diabetes (T2DM) (López‐Otín et al. 2023). As population aging continues to sweep across China and the world, the rising burden of NCDs has become a major global concern (Jiang and Feng 2022). Identifying biomarkers for aging and potential influencing factors is essential for developing strategies to promote healthy aging.

The gut microbiota plays a pivotal role in healthy aging due to its response to environmental signals and its involvement in host immunity, nutrition, metabolism, gut endocrine function, and neurological signaling (López‐Otín et al. 2023; Xiao et al. 2024). Recent studies have documented diverse aging patterns of the gut microbiome, including depletion of microbial diversity, reduction in core taxa and beneficial short‐chain fatty acid (SCFA) producers, and enrichment of pro‐inflammatory pathobionts in the elders (Bradley and Haran 2024). Additionally, increases in the uniqueness and evenness of the gut microbiota have been linked to host age and used to predict health status independently of age (Pang et al. 2023; Wilmanski et al. 2021).

Despite these findings, the literature on age‐related microbial taxa shows considerable inconsistency (Badal et al. 2020). For instance, several studies have indicated that genera such as *Bifidobacterium*, *Clostridium*, and *Faecalibacterium* were more abundant in the elders, while others have noted reduced abundances of these bacteria in aging populations (Badal et al. 2020; Maffei et al. 2017; Salazar et al. 2023). The inconsistency may be due to the pitfalls of the widely used taxon‐based method, which treats bacteria strains within the same taxon as a single variable. This approach may have led to null or spurious associations, as bacteria strains within a single taxon may exert opposing effects on human health and aging (Arboleya et al. 2016). It has been recognized that bacteria in the gut ecosystem interact with each other, forming local networks and functioning as coherent functional groups called “guilds” (Wu et al. 2021). Members of the same guild demonstrate co‐abundant behaviors under environmental perturbations and achieve certain biological functions, making guilds ecologically meaningful indicators of host phenotypes (Wu et al. 2021). In our previous study, we constructed microbial co‐abundance networks and guilds in a Chinese population and demonstrated the advantages of guild‐based analysis over the taxon‐based method (Fu et al. 2025). Thus, the guild‐based approach may also facilitate the identification of age‐related gut microbiota as biomarkers for healthy aging.

The natural intrinsic changes in the microbial ecosystem during aging are influenced by various factors, including sex, lifestyle factors, diseases, and medications (Van Hul et al. 2024). Sex is a key factor shaping the composition and functionality of the gut microbiota, though the mechanisms underlying these changes remain inadequately elucidated (Valeri and Endres 2021). Experimental investigations using animal models and in vitro approaches have revealed that sex hormones are intricately linked to certain gut bacteria, such as *Clostridium perfringens* and *Eubacterium eligens* . These bacteria can produce enzymes like β‐glucuronidase, which are involved in the metabolism of estrogens and testosterone (Colldén et al. 2019; Ervin et al. 2019). Zhang et al. (2021) observed significant sex differences in gut microbial composition across sage groups and revealed sex‐specific aging trajectories of gut microbiota in multiethnic populations. de la Cuesta‐Zuluaga et al. (2019) found that women had a higher predicted microbial age than men in a young and middle‐aged population. Many age‐related diseases are linked to gut microbiota dysbiosis. The presence of NCDs as concomitants of aging may serve as influencing factors or represent outcomes of a perturbed gut microbiota (Ghosh et al. 2022). It has been reported that individuals with metabolic diseases had a different aging trajectory of gut microbiota compared to their healthy counterparts (Fu et al. 2023). The dynamic nature of the gut microbiome, evolving over time in response to various factors, highlights the difficulty and importance of understanding its complex interactions during aging.

In this study, we investigated the sex‐specific aging patterns of gut microbiota using guilds previously constructed based on 16S rRNA gene sequencing data in middle‐aged and elderly Chinese adults. Specifically, we identified sex‐specific microbial signatures associated with age, estimated sex‐specific microbial age, examined its associations with residual life expectancy and prevalent major NCDs, evaluated its prospective association with the risk of T2DM, and explored the potential impact of healthy lifestyles on microbial age. The results may provide insights into sex‐specific microbial phenotypes of aging and contribute to the development of effective strategies for healthy aging.

## 2 Results

### 2.1 Outline of the Study

**FIGURE 1:**
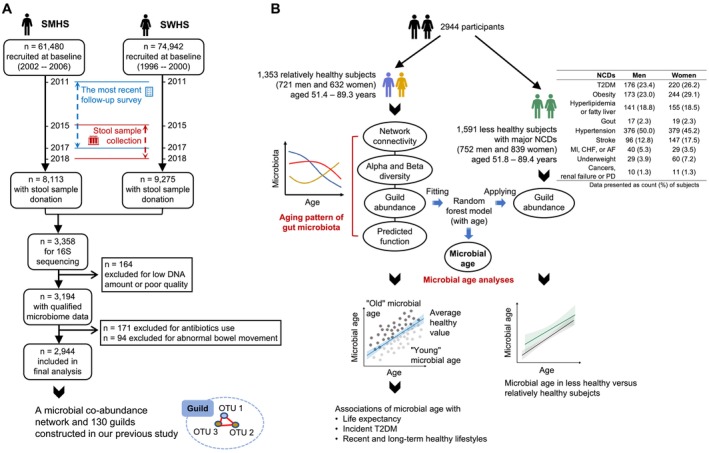
Outline of the study. (A) Recruitment of 2944 participants of the Shanghai Men's and Women's Health Studies. (B) Data analysis strategy of the study.

Figure 1 shows the study outline based on the Shanghai Men's (SMHS) and Women's Health Studies (SWHS). In the first part (Figure 1A), we presented the timeline of the SWHS and SWHS, the recruitment of 2944 participants, and the establishment of 130 microbial guilds in our previous study (Fu et al. 2025). In the second part (Figure 1B), we described the data analysis strategy. Specifically, we evaluated the cross‐sectional associations of guild‐level gut microbiota (network connectivity, diversity, relative abundances, and predicted functions) with chronological age in relatively healthy men and women free of major chronic diseases. Using the identified age‐related guilds, we estimated the average healthy microbial age in men and women by fitting sex‐specific random forest (RF) models among 721 relatively healthy men and 632 relatively healthy women. We then applied the established RF models to less healthy men and women to derive and compare their microbial age with those of their healthy counterparts. We also classified our subjects into subgroups with a “young” or an “old” microbial age based on deviations from the average healthy microbial age and profiled their residual life expectancy across chronological ages. Finally, we prospectively evaluated the associations of microbial age with the risk of T2DM and between long‐term healthy lifestyles and microbial age.

### 2.2 Characteristics of Study Participants

A total of 2944 subjects with qualified fecal microbiome 16S rRNA (V4 region) sequencing data were included. The 1473 men from the SMHS were aged 40–75 years at baseline during 2002–2006, and 51–89 years at stool donation during 2015–2018. The 1471 women from the SWHS were aged 40–71 years at baseline during 1996–2000, and 56–89 years at stool collection. As shown in Table 1 and Figure 1B, over half of the subjects (1591, 54.0%) reported or were registered with metabolic disorders (T2DM, obesity, hyperlipidemia, fatty liver, and gout), cardiovascular disorders (hypertension, stroke, myocardial infraction, congestive heart failure, and atrial fibrillation), underweight, renal failure, Parkinson's disease (PD), or cancers. These individuals were classified as less healthy subjects. To enhance statistical power in subsequent association analysis, we categorized the individuals diagnosed with hyperlipidemia or fatty liver into the “FattyDis” group, and those with myocardial infarction (MI), congestive heart failure (CHF), or atrial fibrillation (AF) into the “HeartDis” group.

**TABLE 1:** Characteristics of study participants by health status.

	All subjects			Men			Women		
	Relatively healthy (*n* = 1353)	Less healthy (*n* = 1591)	*p*	Relatively healthy (*n* = 721)	Less healthy (*n* = 752)	*p*	Relatively healthy (*n* = 632)	Less healthy (*n* = 839)	*p*
Age at baseline survey (years)	53.3 ± 9.1	56.2 ± 9.0	< 0.001	55.0 ± 9.0	57.5 ± 9.6	< 0.001	51.5 ± 8.9	55.0 ± 8.4	< 0.001
Age at sample collection (years)									
Mean ± SD	68.4 ± 9.1	71.8 ± 9.1	< 0.001	67.6 ± 9.1	70.3 ± 9.6	< 0.001	69.4 ± 9.0	73.1 ± 8.5	< 0.001
Range	51.4–89.3	51.8–89.4		51.4–87.0	51.8–88.7		56.1–89.3	56.0–89.4	
Age group (years)									
50–59	259 (19.1)	159 (10.0)	< 0.001	171 (23.7)	114 (15.2)	< 0.001	88 (13.9)	45 (5.4)	< 0.001
60–69	568 (42.0)	556 (34.9)		283 (39.3)	268 (35.6)		285 (45.1)	288 (34.3)	
70–79	311 (23.0)	482 (30.3)		169 (23.4)	206 (27.4)		142 (22.5)	276 (32.9)	
≥ 80	215 (15.9)	394 (24.8)		98 (13.6)	164 (21.8)		117 (18.5)	230 (27.4)	
Educational level									
Primary school or below	144 (10.6)	279 (17.5)	< 0.001	43 (6.0)	53 (7.0)	0.156	101 (16.0)	226 (26.9)	< 0.001
Junior high school	541 (40.0)	582 (36.6)		257 (35.6)	258 (34.3)		284 (44.9)	324 (38.6)	
High or vocational school	571 (42.2)	584 (36.7)		340 (47.2)	330 (43.9)		231 (36.6)	254 (30.3)	
College or above	97 (7.2)	146 (9.2)		81 (11.2)	111 (14.8)		16 (2.5)	35 (4.2)	
Body mass index (kg/m^2^)	23.4 ± 2.2	25.1 ± 4.1	< 0.001	23.6 ± 2.1	25.1 ± 3.7	< 0.001	23.1 ± 2.2	25.2 ± 4.3	< 0.001
Energy intake (kcal/day)	2026 ± 540	2037 ± 565	0.586	2135 ± 554	2166 ± 596	0.292	1902 ± 497	1921 ± 507	0.463
Most recent lifestyle behaviors									
Healthy diet	720 (53.2)	889 (55.9)	0.159	366 (50.8)	397 (52.8)	0.467	354 (56.0)	492 (58.6)	0.339
Regular exercise	692 (51.1)	895 (56.3)	0.006	364 (50.5)	413 (54.9)	0.099	328 (51.9)	482 (57.4)	0.039
Smoking	140 (10.3)	152 (9.6)	0.512	139 (19.3)	152 (20.2)	0.701	1 (0.2)	0 (0.0)	
Regular alcohol drinking	229 (16.9)	229 (14.4)	0.066	217 (30.1)	213 (28.3)	0.490	12 (1.9)	16 (1.9)	1.000
Most recent healthy lifestyle index									
0	12 (0.9)	20 (1.3)	0.005	12 (1.7)	20 (2.7)	0.163	0 (0.0)	0 (0.0)	0.171
1	101 (7.5)	93 (5.8)		98 (13.6)	91 (12.1)		3 (0.5)	2 (0.2)	
2	396 (29.3)	398 (25.0)		245 (34.0)	223 (29.7)		151 (23.9)	175 (20.9)	
3	520 (38.4)	624 (39.2)		236 (32.7)	260 (34.6)		284 (44.9)	364 (43.4)	
4	324 (23.9)	456 (28.7)		130 (18.0)	158 (21.0)		294 (46.5)	298 (35.5)	

*Note:* Data presented as count (%) or mean ± SD. Less healthy subjects defined as those with type 2 diabetes (T2DM), obesity, hyperlipidemia, fatty liver, gout, hypertension, stroke, myocardial infarction (MI), chronic heart failure (CHF), atrial fibrillation (AF), underweight, cancer, renal failure or Parkinson's diseases (PD) at stool sample donation. *p* values for *t* test, *χ* ^2^ test, or Fisher exact test.

### 2.3 Age‐Related Shifts in Microbial Network Connectivity

**FIGURE 2:**
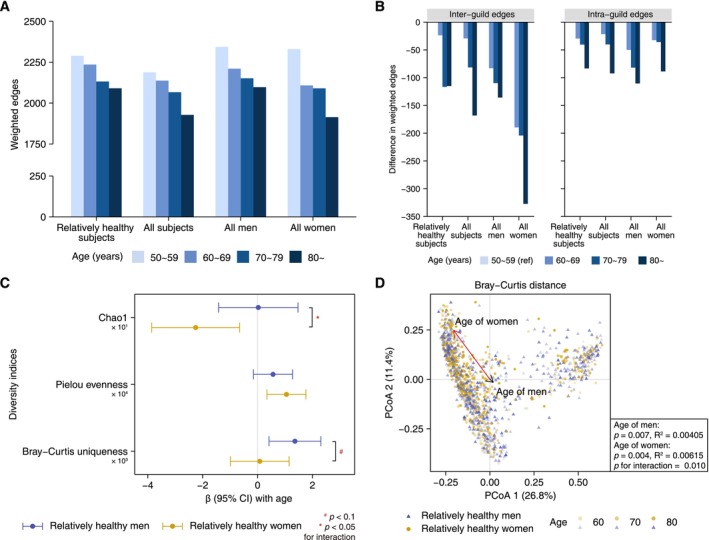
Network connectivity and diversity of gut microbiota at the guild level with age in men and women. (A) Bar plots illustrating network connectivity (weighted edges) derived from microbial co‐abundance networks in four age groups, stratified by sex and health status. (B) Bar plots showing differences in interguild and intraguild weighted edges between age groups, using the youngest age group (50–59 years) as the reference. (C) Forest plot for associations of guild‐level Chao1 richness, Pielou evenness, and Bray–Curtis uniqueness with age in relatively healthy men and women. Points and lines indicate *β* coefficients and 95% confidence intervals of diversity indices with age, derived from multivariate linear models, with ^#^ *p* < 0.10 and *p < 0.05 for the interaction between age and sex. (D) Two‐dimensional PCoA diagram of guild‐level Bray–Curtis distance. Arrows represent regression coefficients of the scores of axis 1 and axis 2 with age, with lengths rescaled to match the coordinates. *p* values and *R* ^2^ values were derived from permutational multivariate analysis of variance. In panels (C) and (D), multivariate analyses were adjusted for BMI and total energy intake.

A total of 130 co‐abundance groups, referred to as microbial guilds, were clustered based on the abundance correlations among 1477 operational taxonomic units (OTUs) from all 2944 subjects (Figure 1A), as previously described (Fu et al. 2025). In the overall co‐abundance network of 1477 OTUs, a total of 3832 edges and 2045.1 weighted edges were identified, with 1567.1 (76.6%) of the weighted edges being intraguild (Table S1). Compared to younger age groups, older age groups exhibited fewer weighted edges (Figure 2A), particularly in the interguild edges (Figure 2B), suggesting a decline in interactions between bacteria from different guilds as age progresses. Moreover, using normalized variation of information (NVI) to assess the similarity of guild structures between subgroups, we obtained similarity scores (1 − NVI) exceeding 0.67 between the four age groups of 50–59, 60–69, 70–79, and ≥ 80 years in both sexes (Table S2), indicating high similarity and stability in the guild structures across age ranges.

### 2.4 Age‐Related Changes in Microbial Guild Diversity in Relatively Healthy Men and Women

In the multivariable analyses of age‐related gut microbiota, we adjusted for body mass index (BMI) and total energy intake as covariates. These factors were significantly associated with overall microbial community structure (i.e., *β*‐diversity) in univariate permutational multivariate analysis of variance (PERMANOVA) stratified by sex (Table S3). Further analysis revealed a decreasing Chao1 index with age in women but not in men (*p* for interaction = 0.040). Specifically, the *β* (95% confidence interval, CI) for each year of age was −0.23 (−0.39, −0.07) in women (Figure 2C). The Pielou evenness increased with age in both sexes, with a significant increase in women (*β*: 1.1E−4, 95% CI: 3E−5, 1.8E−4). Additionally, we observed a rise in Bray–Curtis uniqueness with age, which reached significance only in men (*p* for interaction = 0.084).

To visualize the structural shifts in microbial guilds with age, we performed principal coordinate analysis (PCoA) based on Bray–Curtis dissimilarity. As shown in Figure 2D, the rescaled linear regression coefficients of PCoA axis scores with age were depicted as driving arrows. The longer arrow in women indicated a more pronounced shift in Bray–Curtis distance with age (PERMANOVA *p* for sex heterogeneity = 0.010). This suggests that gut microbiota guilds explained more variance related to age in women (*R* ^2^ in women = 0.00615) compared to men (*R* ^2^ in men = 0.00405). Given that the first two axes (PCoA 1 and PCoA 2) accounted for only 38.2% of the total variance in both men and women, we further examined the top 25 axes (cumulative explained variance > 95%) and observed sex‐specific changes in gut microbiota composition during aging (Table S4).

To test the robustness of our results, we conducted a series of sensitivity analyses. Specifically, we adjusted for additional potential confounding factors, including educational level, regular exercise, smoking, and regular alcohol drinking, in the multivariate analysis of microbial diversity (Figure S1A,B). We also accounted for the time of sample collection and fasting hours prior to sampling (Figure S1C,D). In these analyses, the results remained largely unchanged. Furthermore, we excluded participants who reported using antipyretic analgesics (*n* = 14) or hormone medications (*n* = 4) and did not observe substantially changed results (Figure S1E,F). Collectively, these sensitivity analyses provide support for the robustness of our findings from the main analyses.

### 2.5 Age‐Related Guilds in Relatively Healthy Men and Women

**FIGURE 3:**
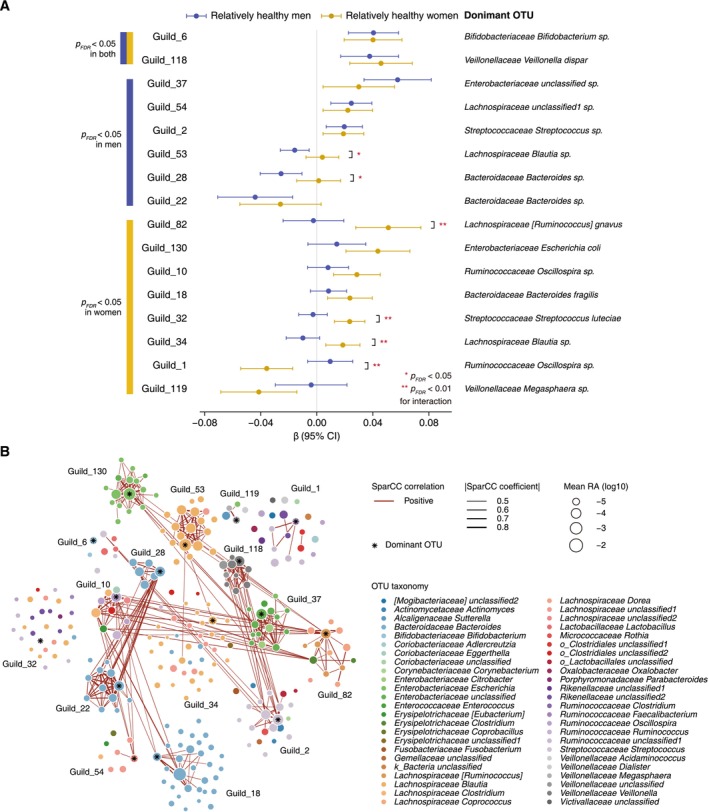
Associations between gut microbiota guilds and age in relatively healthy men and women. (A) Forest plot showing *β* coefficients and 95% confidence intervals of clr‐transformed abundances of microbial guilds with age. The results were derived from multivariate linear models adjusted for BMI and total energy intake. Asterisks denote significance levels for the interaction between age and sex (*p_FDR_ < 0.05 and **p_FDR_ < 0.01). (B) Co‐abundance network of 270 OTUs across 16 identified guilds based on SparCC correlations (*p* < 0.05 and |coefficients| > 0.4). Mean relative abundances of OTUs were calculated using log‐transformed values, with a pseudo‐count of 0.5 added to zero values.

A total of 24 age‐related guilds were identified in men (*p* < 0.05, Table S5) and 27 in women (*p* < 0.05, Table S6), of which 16 were statistically significant after controlling for the false discovery rate (FDR) at 5% (Figure 3). As shown in Figure 3A, in addition to Guild_6 and Guild_118, the only two significant guilds positively associated with age in both sexes, we identified three increasing guilds (Guild_37, Guild_54, and Guild_2) and three decreasing guilds (Guild_53, Guild_28, and Guild_22) exclusively in men. In women, we identified six enriched guilds (Guild_82, Guild_130, Guild_10, Guild_18, Guild_32, and Guild_34) and two reduced guilds (Guild_1 and Guild_119), all with *p*_FDR_ < 0.05. Significant sex heterogeneity was observed for Guild_53, Guild_28, Guild_82, Guild_32, Guild_34, and Guild_1 (all *p*_FDR_ for interaction < 0.05).

Among the significantly elevated guilds, several incorporated bacteria that have been previously associated with aging (Kong et al. 2019; Sun et al. 2023; Wu et al. 2022; Xu et al. 2019; Zhanbo et al. 2024). Specifically, Guild_6 was dominated by *Bifidobacterium* sp. and included *Streptococcus* sp. and all three OTUs of *Lactobacillus*; Guild_118 had *Veillonella dispar* as core bacteria; Guild_37 was dominated by *Escherichia coli* , while Guild_130 was dominated by *unclassified Enterobacteriaceae*, with all OTUs belonging to this family; Guild_34 was dominated by *Blautia*, a genus of *Lachnospiraceae*, while six guilds (Guild_10, Guild_32, Guild_34, Guild_53, Guild_54, and Guild_82) included other members of *Lachnospiraceae* (Table S1).

Figure 3B depicts the co‐abundance network of 270 OTUs that constructed the 16 significant age‐related guilds. These guilds were taxonomically heterogeneous and exhibited positive correlations with each other, particularly with significant interguild correlations between Guild_130 and Guild_37, Guild_28 (*Bacteroides* sp. dominated) and Guild_22 (*Bacteroides* sp. dominated) (all OTUs belonging to *Bacteroides*), Guild_118 and Guild_2 (*Streptococcus* sp. dominated), as well as Guild_82 ( *Ruminococcus gnavus* dominated) and Guild_10 (*Oscillospira* sp. dominated).

### 2.6 Age‐Related Predicted Microbial Functions in Relatively Healthy Men and Women

**FIGURE 4:**
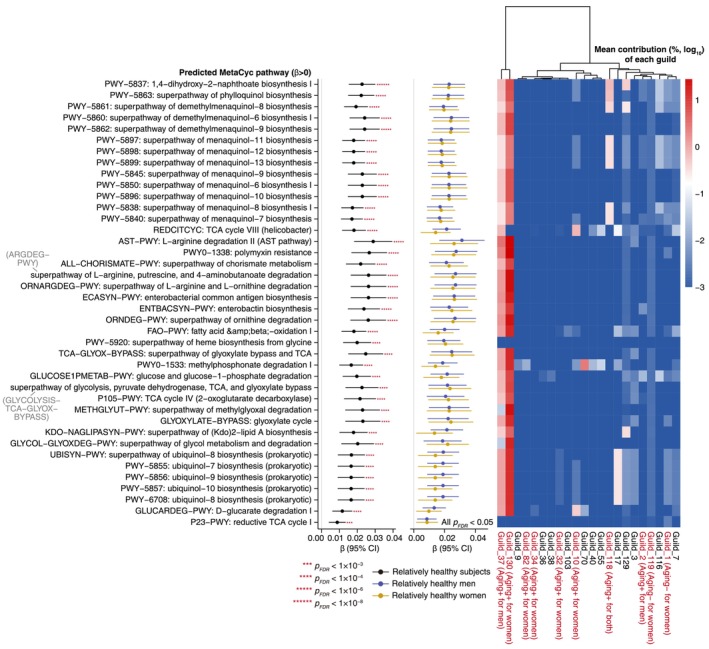
Predicted microbial functions associated with age in relatively healthy men and women. Forest plot presents *β* coefficients and 95% confidence intervals of clr‐transformed abundances of predicted MetaCyc pathways with age. The results were derived from multivariate linear models adjusted for BMI and total energy intake. Heatmap illustrates the mean contribution of each microbial guild to the abundance of predicted pathways. The contribution (%) was log_10_ transformed, with a pseudo‐count of 0.001% added to zero values. Guilds identified as positively or negatively associated with age were noted as “Aging^+^” or “Aging^−^,” respectively.

As shown in Table S7, a total of 172 MetaCyc pathways were significantly related to age in both sexes, including 40 positively related pathways and 132 negatively related pathways (all *p*_FDR_ < 0.05), and no pathways exhibited significant sex heterogeneity. Therefore, we further examined the associations between these pathways and age in the combined population. Interestingly, while the pathways negatively related to age were poorly explained by the identified age‐related guilds (Figure S2), those positively related to age could be primarily attributed to the identified age‐related Guild_37 and Guild_130 (Figure 4). Specifically, the 1,4‐dihydroxy‐2‐naphthoate biosynthesis pathway (PWY‐5837) served as a core route shared by subsequent biosynthesis super‐pathways for menaquinol‐6–13 (PWY‐5850/5840/5838/5845/5896–5899), demethylmenaquinol‐6/8/9 (PWY‐5860–5862), and phylloquinol (PWY‐5863) (Figure 4 and Figure S3).

### 2.7 Microbial Age in Relatively Healthy Men and Women

Considering the sex heterogeneity of gut microbiota, we developed sex‐specific RF models to estimate microbial age using the identified 24 age‐related guilds in men (*p* < 0.05, Table S5) and 27 in women (*p* < 0.05, Table S6). The importance of each included guild predictor is detailed in Table S8, aligning with the magnitude of the associations between the guilds and chronological age presented in Figure 3A. The detailed model performance metrics are listed in Table S9.

**FIGURE 5:**
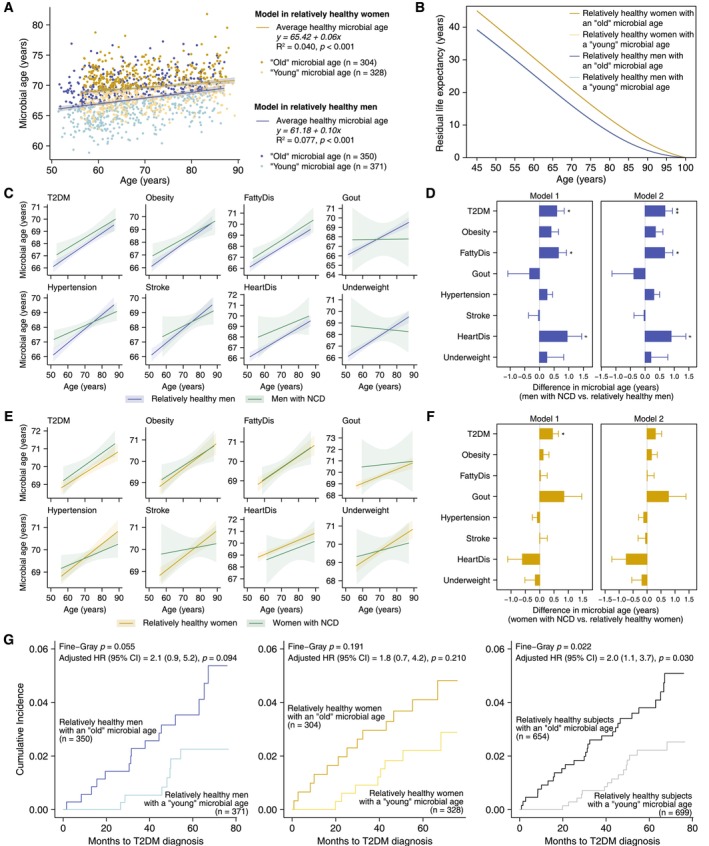
Microbial age and its associations with health status in men and women. (A) Scatter plots of chronological age versus microbial age derived from sex‐specific Random Forest (RF) models for men and women, with linear fit lines representing the average healthy microbial age. Individuals whose microbial age is above or below the curves were categorized into subgroups characterized by an “old” or a “young” microbial age, respectively. The formulas and parameters for the linear fit lines were presented above the plot. (B) Comparison of residual life expectancy between subgroups with an “old” or a “young” microbial age in both sexes. Residual life expectancy was estimated as the area under the survival curve up to 100 years old using flexible parametric survival models, conditionally on surviving from ages 45 to 100 years old. (C, D) Microbial age in men with or without major chronic diseases. (E, F) Microbial age in women with or without major chronic diseases. In panels (C) and (E), linear fit curves of chronological age with microbial age were plotted, with 95% confidence intervals shaded. In panels (D) and (F), differences in microbial age and their standard errors were assessed using multivariate linear models adjusted for age (Model 1) and additionally for body mass index, total energy intake, education level, regular exercise, smoking, and regular alcohol drinking (Model 2), with asterisks denoting the significance levels (#*p* < 0.10, *p < 0.05, and **p < 0.01). (G) Cumulative incidence curves of type 2 diabetes in relatively healthy subjects with an “old” or a “young” microbial age. The difference in cumulative incidence was tested using the Fine‐Gray model. Hazard ratios (HR) were estimated using a Cox proportional hazard regression model, considering the competing risk of all‐cause death and adjusted for age, sex (only for all subjects), body mass index, total energy intake, education level, regular exercise, smoking, and regular alcohol drinking.

By regressing microbial age to chronological age, we derived linear formulas of *y* = 61.18 + 0.10*x* (*R* ^2^ = 0.077, *p* < 0.001) for men and *y* = 65.42 + 0.06*x* (*R* ^2^ = 0.040, *p* < 0.001) for women (Figure 5A). These equations were utilized to calculate the corresponding average healthy microbial age. Based on the deviations between microbial age and these average values, subjects were categorized into subgroups with a “young” (371 men and 328 women) or an “old” microbial age (350 men and 304 women). As shown in Figure 5A, women generally exhibited a higher microbial age than men across the entire age range. Despite this higher microbial age, women had a longer residual life expectancy compared to men throughout the entire spectrum of chronological age (Figure 5B). Additionally, regardless of sex, there was a considerable overlap in residual life expectancy among individuals with either a “young” or an “old” microbial age.

### 2.8 Age‐Related Guilds and Microbial Age by Health Status

We compared the abundances of the 24 age‐related guilds in men and the 27 in women by the presence of NCDs. The directions of the differences were mostly in line with those observed in relation to age in both sexes (Figure S4). Specifically, Guild_6, Guild_118, and Guild_2 were significantly more enriched in men with T2DM and FattyDis, while Guild_6, Guild_118, and Guild_37 were significantly more abundant in women with T2DM. These results suggest that age‐related microbial markers may also function as potential indicators for chronic disease risk assessment.

We then applied the established RF models to less healthy men and women. As illustrated in Figure 5C, the linear regression curves depicting microbial age versus chronological age for men with T2DM, FattyDis, or HeartDis were positioned above those for relatively healthy men. This indicates that men with these conditions had a more advanced microbial age, with statistically significant differences for those with T2DM (difference: 0.68; 95% CI: 0.17, 1.19) or FattyDis (difference: 0.67; 95% CI: 0.11, 1.22) (Figure 5D). In women, individuals with T2DM or gout demonstrated a slightly higher microbial age than their healthy counterparts (Figure 5E,F). Meanwhile, the linear regression curves of microbial age versus chronological age for individuals with other NCDs, such as hypertension and stroke, intersected with those for relatively healthy individuals (Figure 5C,E). This pattern suggests that younger patients with these conditions exhibited a higher microbial age compared to their healthy counterparts, while older patients had a lower microbial age relative to relatively healthy individuals. The differences in microbial age by health status were consistent even after excluding individuals who reported using antipyretic analgesics or hormones medications (*n* = 18 in the healthy and *n* = 77 in the diseased group) (Figure S5).

We further evaluated the prospective association between microbial age and the risk of T2DM. A total of 43 subjects (22 men and 21 women) were newly diagnosed with T2DM during a median (interquartile range, IQR) follow‐up time of 61.0 (46.5, 69.7) months. After accounting for the competing risk of all‐cause death, we observed a higher cumulative incidence of T2DM (Fine‐Gray *p* = 0.022) and a higher risk of incident T2DM in subjects with an “old” microbial age. The adjusted hazard ratio (HR) was 2.0 (95% CI: 1.1, 3.7) after controlling age, sex, BMI, total energy intake, educational level, regular exercise, smoking, and regular alcohol drinking (Figure 5G). Each year of age‐matched difference between microbial age and the average healthy values was associated with a 10% (95% CI: 1.01, 1.19) higher risk of T2DM.

To validate the microbial age established at the guild level, we derived microbial age using the dominant OTUs of age‐related guilds (24 in men and 27 in women), all OTUs within these guilds (266 in men and 376 in women), and age‐related genera (27 in men and 25 in women). As illustrated in Figures S6–S8, the differences in microbial age at the OTU or genus levels by prevalent T2DM or FattyDis were smaller than the guild‐based estimates. However, no significant cross‐sectional associations with other NCDs and prospective associations with T2DM were observed for microbial age derived from significant OTUs or genera. These results indicate that the guild‐based microbial age better captured disease‐related features.

### 2.9 Healthy Lifestyle and Microbial Age in Relatively Healthy Men and Women

**FIGURE 6:**
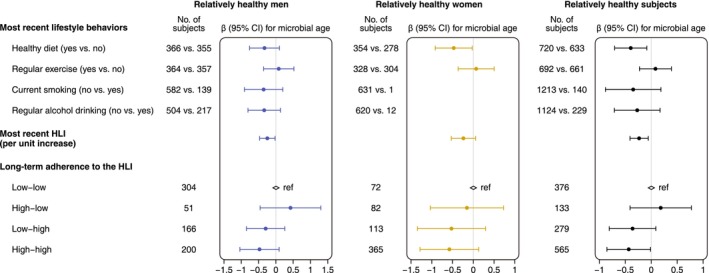
Associations between healthy lifestyles and microbial age in relatively healthy men and women. The points and lines in the forest plot indicate *β* coefficients (95% confidence intervals) of the healthy lifestyle index (HLI) and adherence to a healthy lifestyle with microbial age. The estimates were derived from multivariate linear regression models, adjusted for age, sex (only for all subjects), body mass index, and education level. Long‐term adherence to a healthy lifestyle was assessed by HLI at both baseline and the most recent follow‐up, with an HLI ≤ 2 as a low score and HLI > 2 as a high score.

A lower microbial age was observed in men who maintained a healthy diet or currently abstained from smoking or drinking compared to their counterparts (Figure 6). In women, a significantly lower microbial age was found among those with a healthy diet. A lower microbial age was also noted in all subjects engaging in more healthy behaviors, with each increase in the most recent healthy lifestyle index (HLI) linked to a 0.23‐year (95% CI: −0.41, −0.06) lower microbial age. Regarding the long‐term adherence to HLI, individuals in the high‐high group had the lowest microbial age compared to those in the low‐low group (difference: −0.43; 95% CI: −0.85, −0.01).

## 3 Discussion

In this extensive study of Chinese adults with a wide age range (51–89 years), we elucidated sex‐specific aging patterns of gut microbiota using a guild‐based approach. We observed that microbial network connectivity decreased with age in both sexes, suggesting weaker bacterial interactions and increased vulnerability to external disturbances. Most age‐related microbial changes were sex‐specific, including reduced diversity, increased uniqueness, and altered abundances of microbial guilds. The sex‐specific microbial age, based on identified age‐related guilds, was associated with health status but did not predict residual life expectancy. Importantly, we found significant associations between healthy lifestyle behaviors and microbial age. Our results underscore the interactions between sex and gut microbes during natural aging, expanding our understanding of gut microbiota aging and providing potential strategies for promoting healthy longevity.

Age‐related changes in gut microbiota are part of natural aging and important for maintaining homeostasis. In this study, we found that microbial network connectivity decreased with age, indicating weaker bacterial interactions and a less stable gut microbial ecosystem. This decline is mainly due to two factors. First, the depletion of core bacteria, such as the reduced abundance of *Bacteroides* observed in this study, may lead to a decline in their dominance and loss of connections within the overall network. A similar depletion of core taxa has also been observed among semi‐supercentenarians (Biagi et al. 2016). Second, age‐related intestinal dysfunctions, such as slowed intestinal motility, may hinder the spatial communication of the microbiota. Notably, the majority of decreased network connectivity in older people occurred between guilds, supporting the stability of individual guild structures. Additionally, microbial guild evenness increased with age, consistent with previous findings in centenarians, where increased evenness was associated with youth and longevity (Pang et al. 2023).

We found that gut microbiota diversity decreased and uniqueness increased with age, consistent with the well‐documented decline in microbial diversity during aging (Bradley and Haran 2024; Wilmanski et al. 2021). Most of these age‐related changes were sex specific. The decline in Chao1 richness was more pronounced in women, while the increase in Bray–Curtis uniqueness was more evident in men. Among the 16 significant guilds, 14 exhibited differential abundance in older men or women, with 6 showing significant sex heterogeneity. Only two guilds, Guild_6 (*Bifidobacterium* sp. OTU dominated) and Guild_118 (*Veillonella* sp. OTU dominated), were shared as aging biomarkers in both sexes. These common guilds contained bacteria previously linked to aging (Wu et al. 2022; Xu et al. 2019; Zhanbo et al. 2024), while other sex‐specific guilds with elevated abundance also harbored aging‐related taxa such as *E. coli* , *Enterobacteriaceae* members, *Blautia*, or *Lachnospiraceae* members (Kong et al. 2019; Sun et al. 2023). Several guilds with decreasing abundance contained core bacteria from the genus *Bacteroides*, many species of which are known to degrade intestinal mucus (Glover et al. 2022). The reduced abundance of *Bacteroides* in elderly hosts, characterized by declined mucus secretion from gut epithelial goblet cells, may represent a natural adaptation to maintain the integrity of the mucus layer and intestinal barrier (Wilmanski et al. 2022).

As age increases, both men and women experience a decline in sex hormones. The sex‐specific age‐related changes in gut microbiota may be due to the bidirectional communication between the microbiota and the endocrine system. This interaction involves the direct modulation of microbial metabolism by sex hormones and the reciprocal influence of microbial metabolites on the host (Valeri and Endres 2021). A systematic review revealed that decreased microbial diversity was strongly correlated with lower estrogen levels in women and lower testosterone levels in men (d'Afflitto et al. 2022). The sharper decline in estrogens among postmenopausal women compared to the gradual decrease in testosterones among older men (Zhang et al. 2021) may contribute to the more pronounced decrease in gut microbiota richness observed in women during aging. The elevated abundance of Guild_82 ( *R. gnavus* dominated) in older women is consistent with a previous report, in which serum estradiol levels were negatively correlated with the abundance of *R. gnavus* in obese women with polycystic ovary syndrome (Zhou et al. 2020). However, no evidence was available for the relationship between hormone levels and the abundance of *Blautia*, a bacterium that dominates two guilds (Guild_53 and Guild_34) which were more abundant in aged women but less in aged men. It is proposed that sex hormones may modify gut microbiota during aging, thereby contributing to sex‐specific aging patterns of gut microbiota, potentially through interactions with diet, emotion, and nonsteroid hormones (Valeri and Endres 2021). However, it remains speculative to attribute sex differences exclusively to hormonal changes. Further longitudinal studies incorporating endocrine profiling are needed to elucidate the mechanisms underlying the sex heterogeneity.

Intriguingly, the associations between predicted microbial functions and age were consistent across men and women, particularly for metabolic pathways involved in the production of 1,4‐dihydroxy‐2‐naphthoate (DHNA, a prebiotic), phylloquinol (vitamin K1), and menaquinol (vitamin K3). Vitamin K is renowned for its anti‐inflammatory properties and its role in enhancing bone health in the elderly. It is primarily obtained from food and produced by certain strains of *E. coli* (Shineberg and Young 1976). However, some strains of *E. coli* can produce toxic factors and induce diseases, especially during aging (Croxen and Finlay 2010). Our findings highlight the importance of maintaining healthy intrinsic process in addition to reversing pathogenic alterations in the elderly.

This study enriches evidence that microbial age, based on significant microbial guilds, may reflect an intrinsic aspect of sex‐specific aging in relatively healthy populations. We found that women had a higher microbial age but longer residual life expectancy than men of the same chronological age. However, there was no significant difference in residual life expectancy between individuals with “young” and “old” microbial ages. Meanwhile, a higher microbial age was observed in patients with metabolic disorders and was associated with a higher risk of incident T2DM. The higher microbial age in women compared to men may be due to earlier female puberty onset (de la Cuesta‐Zuluaga et al. 2019). The lack of a significant association between microbial age and longevity in our study might be due to the relatively short follow‐up duration, which limited the capture of mortality outcomes among our healthy participants, especially considering the more than 83 years of life expectancy in Shanghai. Our findings on the associations between microbial age and NCDs are consistent with a recent study linking higher microbial age to increased cardiovascular disease risk (Wang et al. 2024), but conflict with another study showing intersecting microbial age curves for patients with certain conditions and healthy individuals (Fu et al. 2023). This intersecting pattern was also observed in some patients with hypertension, stroke, and underweight in this study, likely due to survival bias in the recruitment of older patients. Since residual life expectancy reflects life length and NCD presence indicates life quality to some extent, our results suggest that microbial age may be more closely related to health status than longevity.

A variety of interventions have been employed to modulate gut microbes, including fecal microbiota transplant (FMT), microbial consortia cultivation, the use of prebiotics, probiotics, postbiotics, and adherence to healthy lifestyles (Wargo 2020). However, caution is needed when considering FMT as a potential intervention for reversing aging processes, despite its promising results in animal studies (Wilmanski et al. 2022). In contrast, adopting healthy lifestyles appears to be the most moderate and accessible approach to shaping gut microbial communities. Encouragingly, individuals who engaged in healthier behaviors had a lower microbial age in our study. A healthy diet, rich in fiber and low in fat, has been shown to enrich SCFA producers and reduce harmful microbes such as *E. coli* , *Streptococcus*, *Clostridium*, and *Bacteroides* (Zhang 2022). Moreover, *Prevotella* sp. in Guild_36 were more abundant in smokers (Antinozzi et al. 2022), while pro‐inflammatory *Enterobacteriaceae* in Guild_37 and Guild_130 are opportunistic pathogens associated with alcohol consumption (Dubinkina et al. 2017). Regular excise was weakly associated with microbial age in our study but was linked to higher abundances of beneficial butyrate‐producing bacteria (Mailing et al. 2019). It is reasonable to suggest that adherence to healthier lifestyles can slow down the aging of gut microbiota and potentially delay other biological aging metrics, such as the phenotypic age, and telomere length reduction (Liu et al. 2024).

The strengths of this study include the use of the guild method, high‐quality survey and sequencing data, and a wide age range of participants, which ensured robust guild construction and accurate association evaluation. However, the interpretation of our results is subject to several limitations. First, although our subjects were from two well‐executed cohort studies, the fact that each participant provided only one stool sample restricted our analysis of longitudinal changes in gut microbiota. Additionally, cross‐sectional evaluations are susceptible to birth cohort effects and the risk of reverse causation. Nonetheless, assessing microbial changes associated with aging in a population with a broad age range is a widely adopted approach in prior research (Ghosh et al. 2022). Second, health status was primarily based on self‐reports, with supplementary information obtained through record linkage with local systems that lacked detailed classification of disease subtypes. This reliance on self‐reported data may have introduced misclassification bias, and thereby diminishing the statistical power to evaluate associations between microbial age and the risks of NCDs other than T2DM. Third, function analysis was limited by the nature of 16S rRNA sequencing data, and the profiles predicted by PICRUSt2 may not accurately reflect microbial metabolisms. Further metagenomic and metabolomic investigations are needed to validate key pathways and provide deeper insights into aging‐related microbial guilds. Moreover, the sample size in prospective analyses was insufficient to yield significant associations between microbial age and T2DM stratified by sex. Larger samples or longer follow‐ups are needed for robust results. Furthermore, the RF models in this study had low explanatory power for chronological age (*R* ^2^: 0.040–0.077), consistent with previous studies (*R* ^2^ < 0.10) (de la Cuesta‐Zuluaga et al. 2019; Fu et al. 2023; Wang et al. 2024). Given that chronological age is easily obtained in clinical and population settings, the primary objective of these studies was not to achieve accurate age prediction, but to construct a composite indicator that reflects age‐related microbial changes. Finally, due to the absence of data required for estimating biological age, the optimal metric for aging, we used chronological age as a proxy. To mitigate the influence of physiological factors on the estimation of microbial age, we selected subjects free of major NCDs as the healthy. Nevertheless, we could not fully eliminate the influence of residual and unmeasured confounders.

In conclusion, this study reveals sex‐specific changes in gut microbiota during aging at the guild level among Chinese men and women. Our findings offer insights into the microbial phenotype of aging and its related functions, highlight the role of multiple major NCDs in accelerating aging, and indicate the importance of a healthy lifestyle in slowing down the aging process in the context of gut microbiota.

## 4 Methods

### 4.1 The SWHS and the SMHS

The SWHS and SMHS are large‐scale population‐based cohort studies in urban Shanghai, initiated in 1996 and 2002, respectively, (Shu et al. 2015; Zheng et al. 2005). The cohorts included 61,480 men and 74,942 women aged 40–75 years at baseline, with the primary aim of investigating the impact of environmental, lifestyle, and genetic factors on cancer and other NCDs.

Baseline data were collected via in‐person interviews by trained retired nurses, covering demographics, anthropometric measurements, smoking and drinking status, dietary habits, physical activity, diagnoses of 21 common diseases, and family history of these diseases at baseline. For lifestyle factors, all participants were asked yes or no questions regarding their regular exercise, current smoking, and regular drinking. Dietary information was collected using validated food‐frequency questionnaires, with nutrient intake calculated based on the China Food Composition Table (6th Edition). The Chinese Food Pagoda (CHFP) score was used to assess dietary habits based on the consumption of grains and tubers, vegetables, fruits, dairy products, legumes, meat and poultry, fish and shrimp, eggs, oils, and salt (Yu et al. 2014). Each of the 10 food components was assigned a maximum and 0 points based on the recommended intakes in the Food Pagoda. The final CHFP score is the cumulative sum of all component scores with a range of 0–45 points.

BMI was calculated as weight (kg) divided by height squared (m^2^). Information was updated in five waves of follow‐up for the SWHS (2000, 2002, 2004, 2007, and 2011) and in three waves for the SMHS (2004, 2008, and 2011) (Figure 1).

The studies were approved by the Institutional Review Boards of the Shanghai Cancer Institute and Vanderbilt University Medical Center, with all participants providing informed consent.

### 4.2 Participants of the Study

The current study included 3358 individuals selected from 17,388 cohort participants who donated fecal samples during 2015–2018. Participants were provided with stool sample collection kits and instructions to collect a peanut‐sized stool into a tube containing 5‐mL 95% ethanol (Wang et al. 2021). At the time of donation, participants completed a questionnaire recording the date and time (morning or afternoon) of collection, fasting hours, recent use of antibiotic and medications (e.g., antihypertensives, hypoglycemics, lipid‐lowering agents, antipyretic analgesics, and hormones in the past 7 days and 6 month), bowel movement frequency, and any recent changes in diet or weight.

Fecal samples were shipped to the laboratory within 24 h of collection, where they were stored in aliquots at −80°C. For the current analysis, we excluded 164 subjects with low DNA amount or poor quality samples, 171 who had used antibiotics in the past 6 months, and 94 with constipation and/or diarrhea in the past 7 days, resulting in a final sample size of 2944 participants.

Health status was assessed based on the presence of major NCDs (Li et al. 2024). Participants were categorized as relatively healthy or less healthy according to diagnoses of T2DM, obesity, gout, hyperlipidemia, fatty liver, hypertension, stroke, MI, CHF, AF, underweight, cancer, renal failure, and PD at the time of fecal sample donation. These conditions are known to be associated with altered gut microbiota (Ghosh et al. 2022). Obesity was defined as BMI ≥ 28.0 kg/m^2^, and underweight as BMI < 18.5 kg/m^2^. Diagnoses were based on self‐reported history and medication use. Prevalent hypertension was supplemented by measured blood pressure, and prevalent T2DM was identified through record linkage with the Shanghai Diabetes Management System, where diagnoses were made by clinicians based on fasting and postprandial blood glucose levels, following the 1999 WHO criteria.

Incident T2DM cases were identified from the Shanghai Diabetes Management System up to 1 October 2021, and all‐cause deaths from the Vital Statistics of Shanghai up to 19 August 2024.

### 4.3 Definition of Healthy Lifestyles

We evaluated a healthy lifestyle based on four criteria: regular exercise, no current smoking, no regular alcohol drinking, and a healthy diet, using data from the baseline and most recent follow‐up surveys of the SWHS and SMHS, in line with the WHO recommendation. A healthy diet was defined as being in the top two quintiles of the CHFP score or having a dietary fiber intake exceeding 20 g/day, given the applicability of the CHFP score in Chinese populations (Yu et al. 2014) and the beneficial effect of dietary fiber on gut microbiota (Koponen et al. 2021).

The HLI was defined as the number of healthy behaviors adopted by participants, with a range from 0 to 4, where a higher index indicates a healthier lifestyle. Participants were categorized into two groups based on their HLI: low score (HLI ≤ 2) and high score (HLI > 2). To assess long‐term adherence to a healthy lifestyle, participants were further stratified into subgroups based on their HLI at baseline and the most recent follow‐up: low‐low (low score at both time points), low‐high (low score at baseline and high score at follow‐up), high‐low (high score at baseline and low score at follow‐up), and high‐high (high score at both time points).

### 4.4 16S rRNA Gene Sequencing and Microbial Guild Construction

Stool samples were sequenced for the V4 region of the 16S rRNA gene using Illumina HiSeq (Wang et al. 2021). The resulting clean reads were clustered into OTUs at a sequence identity threshold of 97%. Each OTU was taxonomically assigned against the Greengenes database, yielding an OTU table comprising 14,701 annotated or unannotated OTUs. These OTUs were used to construct microbial guilds following the procedure recommended by Wu et al. (2021).

As described in our previous report (Fu et al. 2025), 1477 OTUs occurring in at least 20% of the 2944 participants were used to build a co‐abundance network using Sparse Correlations for Compositional Data (SparCC). Network connectivity metrics, including degree/edge and weighted degree/edge, were calculated based on SparCC correlations with a *p* value < 0.05 and an absolute correlation coefficient value > 0.4 using the R package *igraph* (version 1.3.5). The correlation matrix was converted into a distance matrix (1 − correlation coefficient) and subsequently clustered using Ward's algorithm. The dissimilarities between clades of the clustering tree were tested by PERMANOVA as the clustering height decreased. A total of 130 heterogeneous clades were regarded as microbial guilds (Table S1). For each guild, the OTU with the highest weighted degree or, in cases where the degree was zero, the OTU with the highest mean relative abundance was designated as the dominant OTU. Intraguild and interguild connectivity metrics were further distinguished based on the structure of the 130 guilds.

The stability of the guild structure was proved by replicating the guild construction procedure across different health statuses (Fu et al. 2025). Further validation was conducted by constructing guilds by sex and then by age groups (50–59, 60–59, 70–79, and ≥ 80 years). The NVI was used to assess the similarity of guild structures between subgroups, with higher 1 − NVI values (ranging from 0 to 1) indicating greater similarity. The connectivity of microbial networks among the four age groups was compared using the 50–60 years age group as the reference.

### 4.5 Predicted Function of Microbial Guilds

To understand the potential mechanisms underlying aging, we predicted the functions (MetaCyc pathways) of the 1477 selected OTUs utilizing the PICRUSt2 tool. The mean contribution (%) of each guild to each functional pathway was calculated by averaging the log‐transformed contributions across all samples, with “zero” values replaced by a pseudo‐count of 0.001%.

### 4.6 Statistical Analysis

We rarefied the guild abundance tables to a minimum sequencing depth of 16,332 reads across all 2944 samples and calculated *α*‐diversity (Chao1, Shannon, and Pielou evenness indices) and *β*‐diversity (Bray–Curtis distance) using the R package *vegan*. We also extracted the minimum Bray–Curtis distance for each sample from the dissimilarity matrix to quantify compositional divergence, where higher values indicate a more distinct gut microbiome, which is a healthier trait associated with aging (Wilmanski et al. 2021). We examined age‐related changes in Bray–Curtis dissimilarity using PERMANOVA with 999 permutations, visualized these changes via PCoA, normalized the linear regression coefficients of PCoA axis scores with age, and depicted this relationship as a directional arrow in the plot.

Given the characteristic enrichment and subsequent decline of intestinal microbiota across an individual's lifespan (Bradley and Haran 2024), we investigated the potential nonlinear trajectory of gut microbiota in relatively healthy subjects aged 50–89 years using restricted cubic spline regression. However, no significant nonlinear relationship was identified (data not shown). Consequently, we assumed a linear trajectory for changes in gut microbiota during aging. We employed multivariate linear models with the R package of *MaAsLin2* to assess the associations of *α*‐diversity, Bray–Curtis uniqueness, abundances of guilds and genera, and Metacyc pathways with age among relatively healthy men and women. These analyses were adjusted for potential confounders such as BMI and total energy intake. Sensitivity analyses further adjusted for education, exercise, smoking, alcohol use, sample collection time, and fasting hours. For the center log‐ratio (clr) transformation, all zero values in the abundance table were replaced with a pseudo‐count of 0.5, equivalent to half of the minimum nonzero value. An interaction term of age × sex was incorporated into the models to assess sex‐specific differences.

Subsequently, we built two RF models using the R package *randomForest* in relatively healthy men and women, respectively. The abundances of sex‐specific age‐related guilds were used as features to predict chronological age, with the predicted values being defined as microbial age. To validate this microbial age, we also derived microbial age using dominant OTUs of these age‐related guilds, all OTUs within the guilds, and age‐associated genera, and compared their downstream associations.

To ensure model robustness, we performed 10‐fold cross‐validation across all features and found the lowest mean squared error with at least 20 features. Thus, we incorporated all age‐related guilds or genera (by *p* < 0.05) in relatively healthy men or women without applying FDR correction. To enhance model stability and reduce overfitting, we set the parameter “mtry” to one‐third of the feature count and “ntree” to 1000. These sex‐specific RF models were then applied to men and women with NCDs to obtain their microbial age.

We then conducted separate linear regression analyses in relatively healthy men and women, with microbial age as the dependent variable (*y*) and chronological age as the independent variable (*x*). The regression fit values were used to establish the average healthy microbial age for each sex. Individuals with a microbial age above this average were categorized as having an “old” microbial age (difference > 0), indicating accelerated aging. In contrast, those with a microbial age below the average were classified as having a “young” microbial age (difference < 0).

We used the R package *flexsurv* to estimate residual life expectancy by calculating the area under the survival curve up to 100 years, conditional on surviving from ages 45 to 100 years. The differences in residual life expectancy between subjects with an “old” and “young” microbial age were assessed by comparing these areas.

We further evaluated the differences in microbial age and age‐related guild abundances between relatively healthy and less healthy subjects using three distinct models. Model 1 adjusted for chronological age only. Model 2 added adjustments for BMI, total energy intake, education level, regular exercise, smoking, and alcohol use. Model 3, serving as a sensitivity analysis, further adjusted for antihypertensive, hypoglycemic, or lipid‐lowering agents, and excluded users of antipyretic analgesics or hormones. Additionally, we used multivariate linear models adjusted for chronological age, BMI, and education level to assess microbial age differences between subjects with high and low HLI.

To evaluate the impact of microbial age on T2DM incidence during follow‐up, we performed survival analysis using the R package *tidycmprsk* with Fine and Gray modeling, considering all‐cause mortality as a competing event. We further applied a Cox proportional hazard regression model to adjust for multiple covariates, using the cause‐specific hazard function to account for competing risk. This approach was used to estimate the HR and 95% CI of microbial age (categorized as “old” vs. “young” and assessed as each‐year difference from average healthy values) with the risk of T2DM.

Statistical analyses were conducted using R version 4.2.0. A *p* value < 0.05 was considered statistically significant. Multiple tests were corrected using the Benjamini–Hochberg procedure to control the FDR.

## Author Contributions

W.X. conceived the study; W.Z. and X.‐O.S. designed the cohort studies; W.X., Y.J., and L.W. collected data and samples; J.F. and H.C. analyzed data; J.F. wrote the paper, and all coauthors critically read and revised the manuscript.

## Conflicts of Interest

The authors declare no conflicts of interest.

## Supporting information


**Figures S1–S8:** acel70192‐sup‐0001‐FiguresS1‐S8.pdf.

**Tables S1–S9:** acel70192‐sup‐0002‐TablesS1‐S9.xlsx.

## Data Availability

Questionnaire‐based survey data, raw 16S rRNA sequencing data, metadata information, code book, and all other data will be available upon research study application and approval by the cohort committees. Permission is required to access the data and resources. The policy for data sharing and request procedures can be found at: https://swhs‐smhs.app.vumc.org. The analytic codes are deposited at GitHub under https://github.com/GinxFu/Aging_guild.
