# Supplementary material for: Sex‐Specific Aging Patterns of Gut Microbiota in Urban Chinese Adults: Guild‐Based Analysis and Implications for Healthy Aging
Source: Aging Cell. 2025 Aug 4;24(10):e70192. doi: 10.1111/acel.70192 (PMC12507401; doi:10.1111/acel.70192)
Supplement: Supplementary file 1 — Figures S1–S8: acel70192‐sup‐0001‐FiguresS1‐S8.pdf. [file ACEL-24-e70192-s001.pdf]

Supplementary Figures

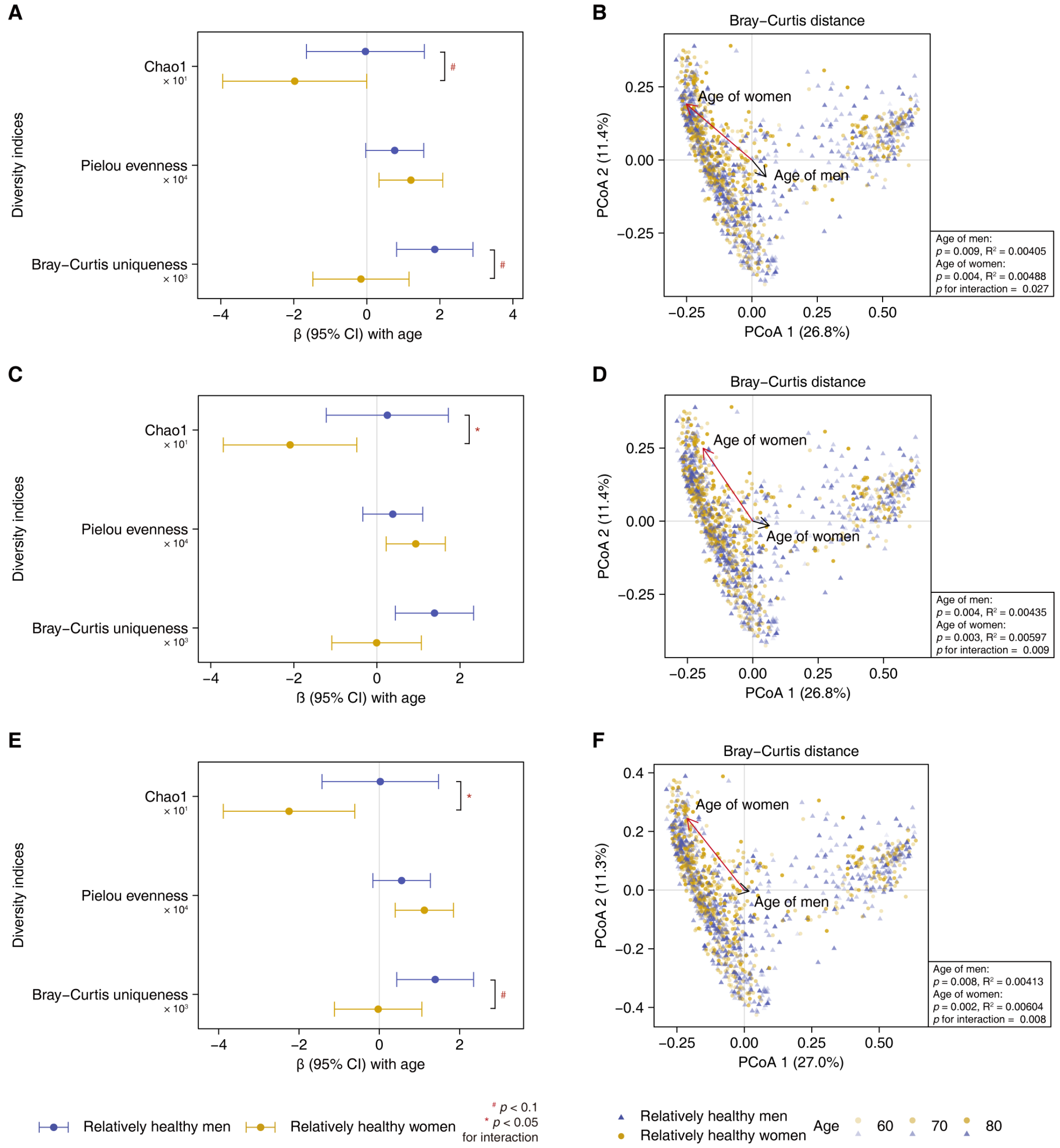

Figure S1 Sensitive analyses of associations between microbial diversity at the guild level and age in relatively healthy men and women. (A, C, and E) Forest plots for the associations between guild-level Chao1, Pielou evenness, and Bray-Curtis uniqueness and age. Points and lines indicate β coefficients and respective 95% confidence intervals of diversity indices with age, derived from multivariate linear models. The interaction between age and sex was shown with $^{\#}p < 0.10$ and $^{*}p < 0.05$. (B, D, and F) Two-dimensional PCoA diagrams of guild-level Bray-Curtis distance. Arrows represent linear regression coefficient of the scores of axis 1 and axis 2 with age, with lengths rescaled to match the coordinates. *p*-values and R^2 were derived from permutational multivariate analysis of variance. Multivariate analyses were adjusted for body mass index, total energy intake, and additionally for education level, regular exercise, smoking, and regular alcohol drinking (panels A and B), as well as time of sample collection and fasting hours (panels C and D). In panels (E) and (F), participants who reported using antipyretic analgesics (n = 14) or hormone medications (n = 4) were excluded.

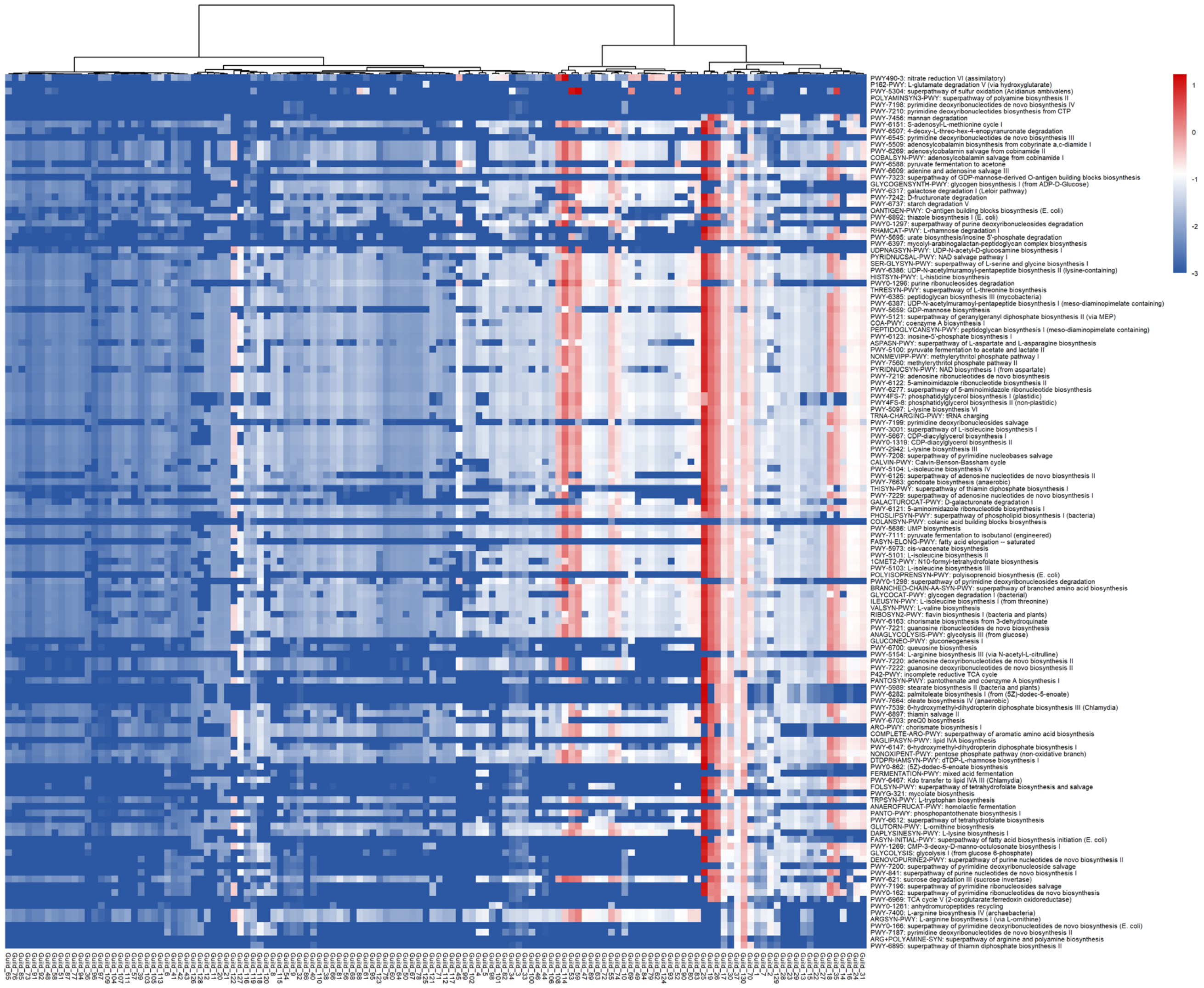

Figure S2 Heatmap of mean contribution of each guild to the abundance of predicted metabolic pathways negatively associated with age. The contribution (%) was $\log_{10}$ transformed, with a pseudo count of 0.001 added to the zero values.

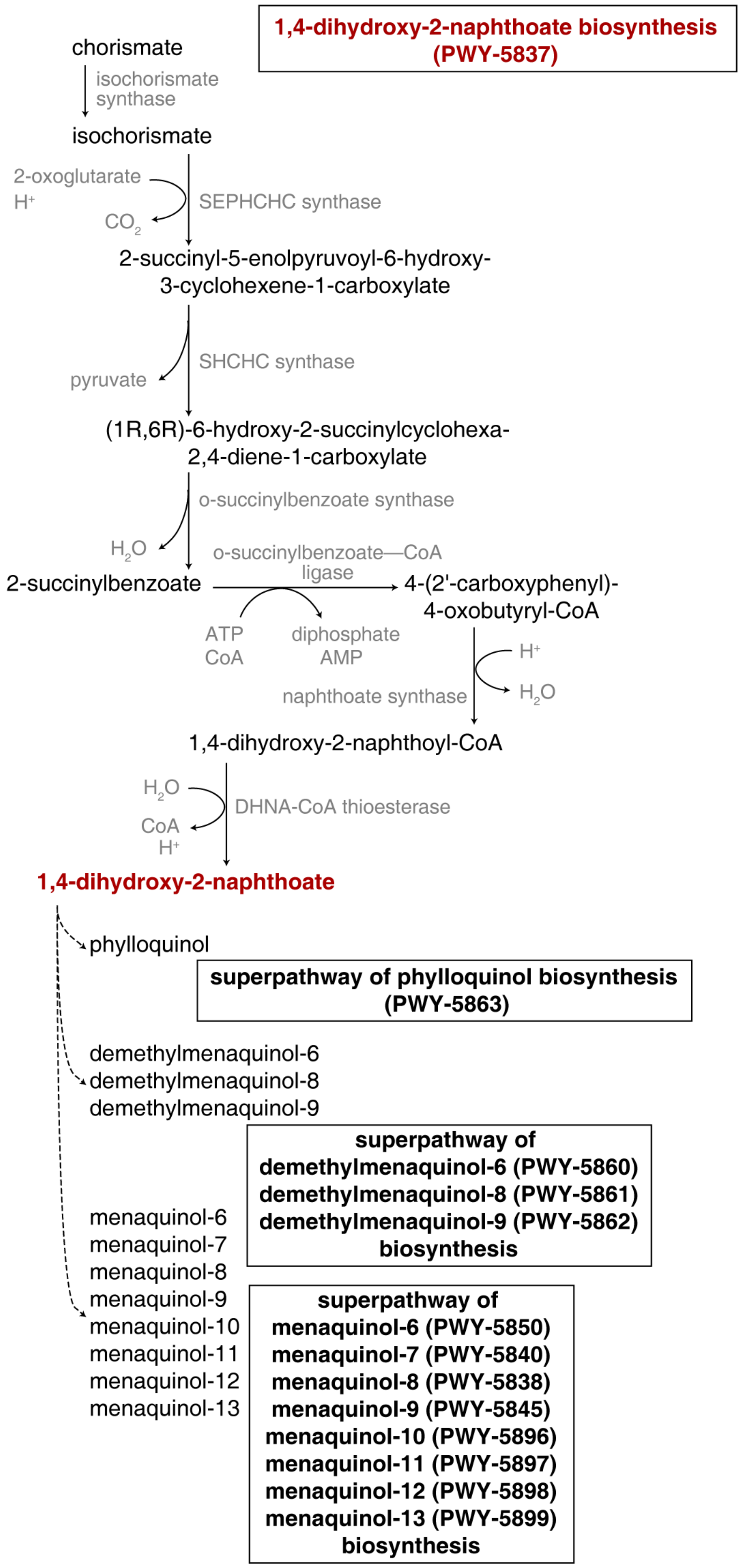

Figure S3 Identified metabolic pathways positively associated with age

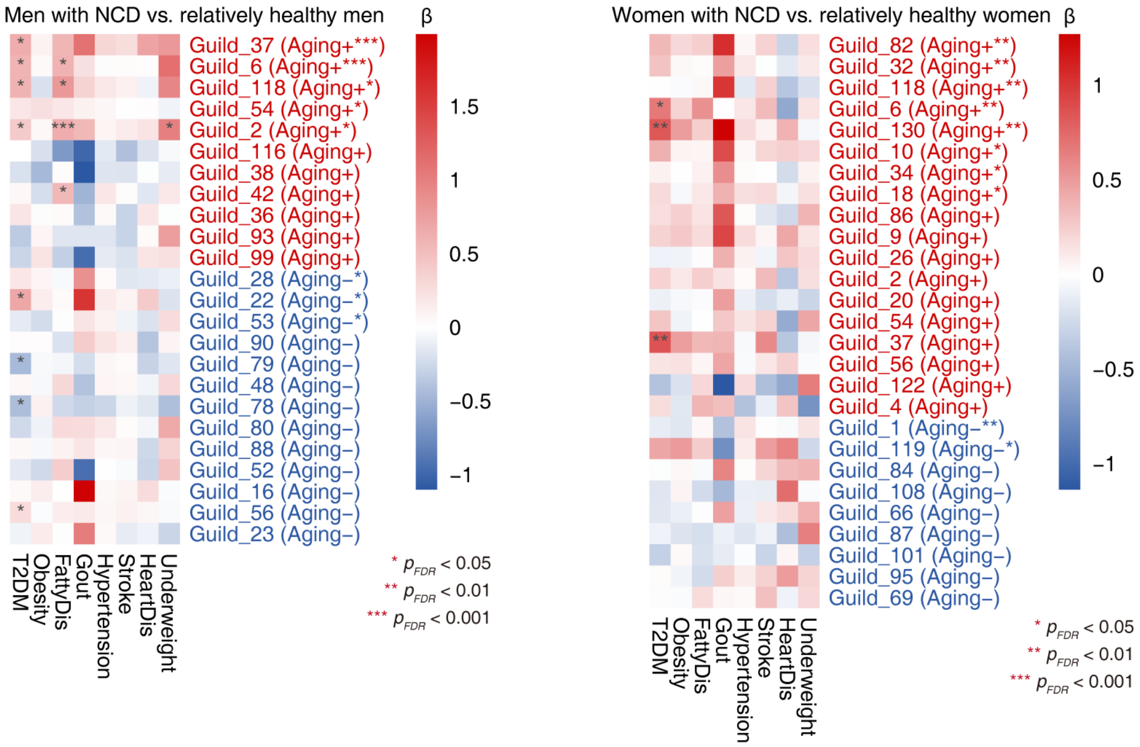

Figure S4 Abundances of age-related guilds by health status in men and women. Heatmap illustrates the differences in clr-transformed abundances of age-related guilds between subjects with or without major chronic diseases. The differences were assessed using multivariate linear models adjusted for age, body mass index, total energy intake, education level, regular exercise, smoking, and regular alcohol drinking. Guilds identified as positively or negatively associated with age were noted as "Aging+" or "Aging−", respectively.

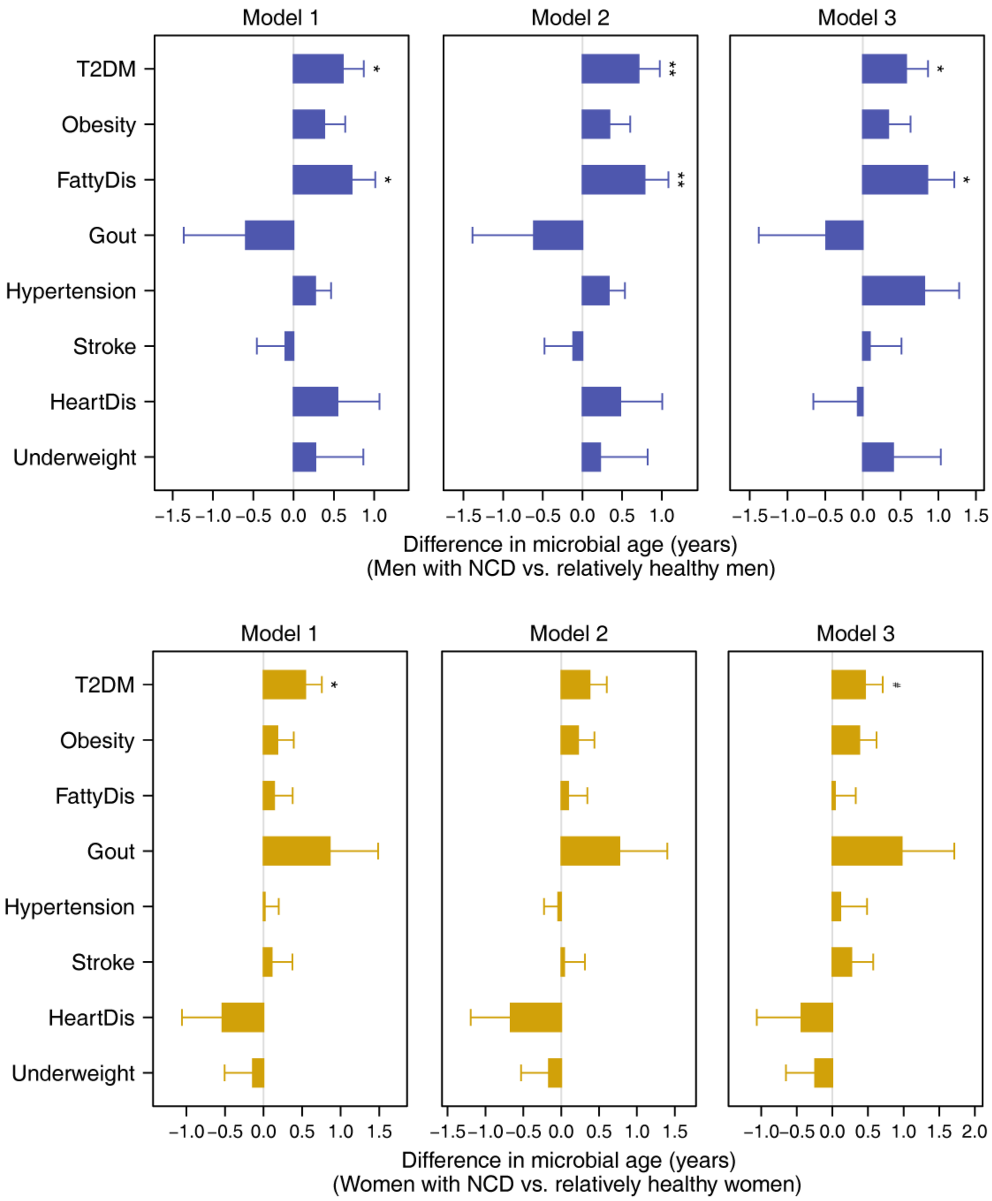

Figure S5 Differences in microbial age by health status in men and women. Participants who reported the use of antipyretic analgesics or hormone medications (18 in the healthy group and 77 in the diseased group) were excluded. Differences in microbial age and their standard errors were assessed using multivariate linear models adjusting for age (Model 1), additionally for body mass index, total energy intake, education level, regular exercise, smoking, and regular alcohol drinking (Model 2), and further for use of antihypertensive, hypoglycemic or lipid-lowering agents (Model 3).

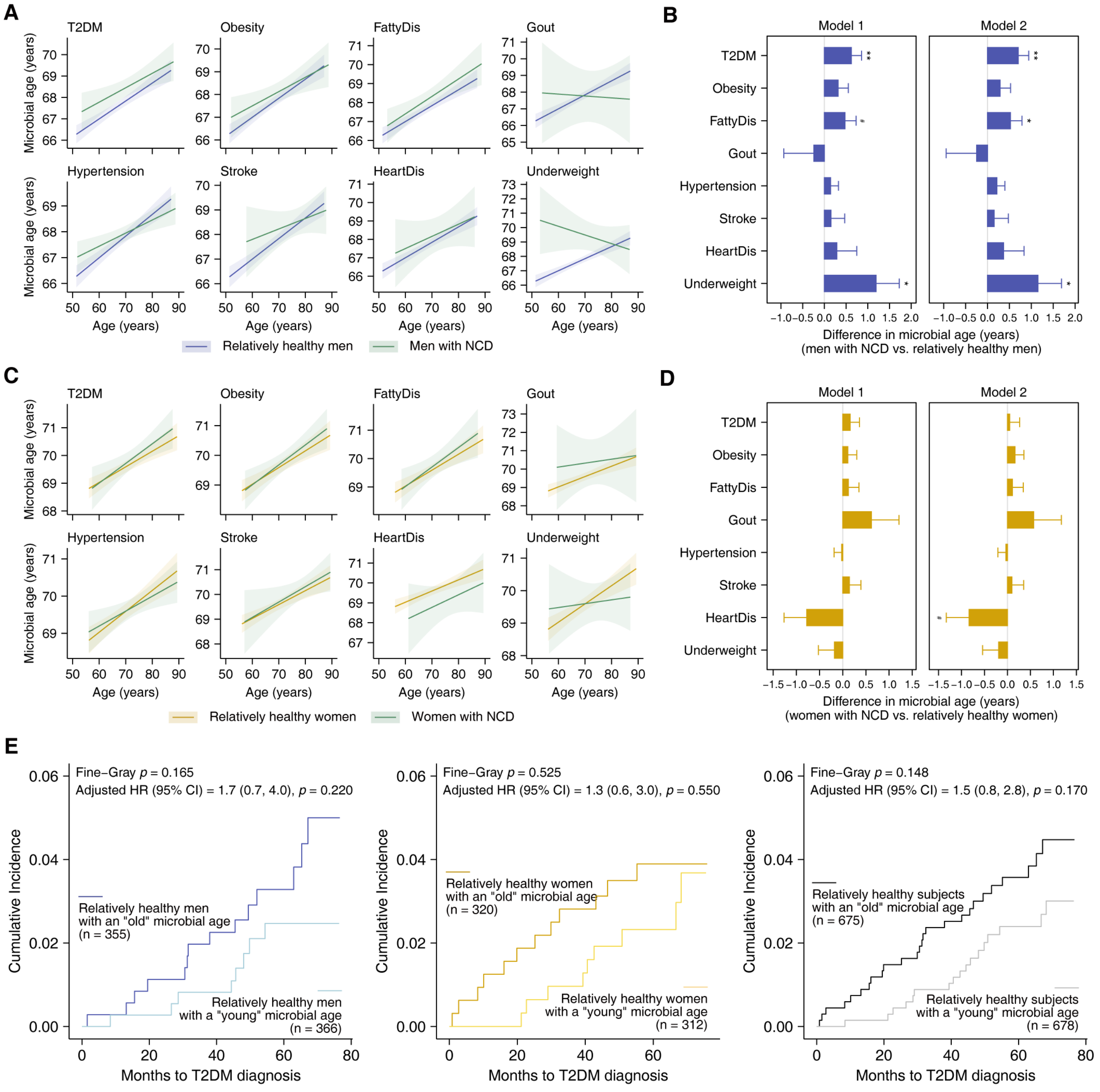

Figure S6 Microbial age estimated based on dominant OTUs of age-related guilds and differences by health status in men and women. (A and B) microbial age in men with or without major chronic diseases, estimated based on 24 dominant OTUs of age-related guilds in men. (C and D) microbial age in women with or without major chronic diseases, estimated based on 27 dominant OTUs of age-related guilds in women. In (A) and (C), linear fit curves of chronological age with microbial age were plotted, with 95% confidence intervals shaded. In (B) and (D), differences in microbial age and their standard errors were assessed using multivariate linear models adjusting for age (Model 1), and additionally for body mass index, total energy intake, education level, regular exercise, smoking, and regular alcohol drinking (Model 2). (E) Cumulative incidence curves of type 2 diabetes in relatively healthy subjects with an "old" or a "young" microbial age. The difference in cumulative incidence was tested using the Fine-Gary model. Hazard ratios (HRs) were estimated using a Cox proportional hazard regression model considering the competing risk of all-cause death and adjusted for age, sex (only for all subjects), body mass index, total energy intake, education level, regular exercise, smoking, and regular alcohol drinking.

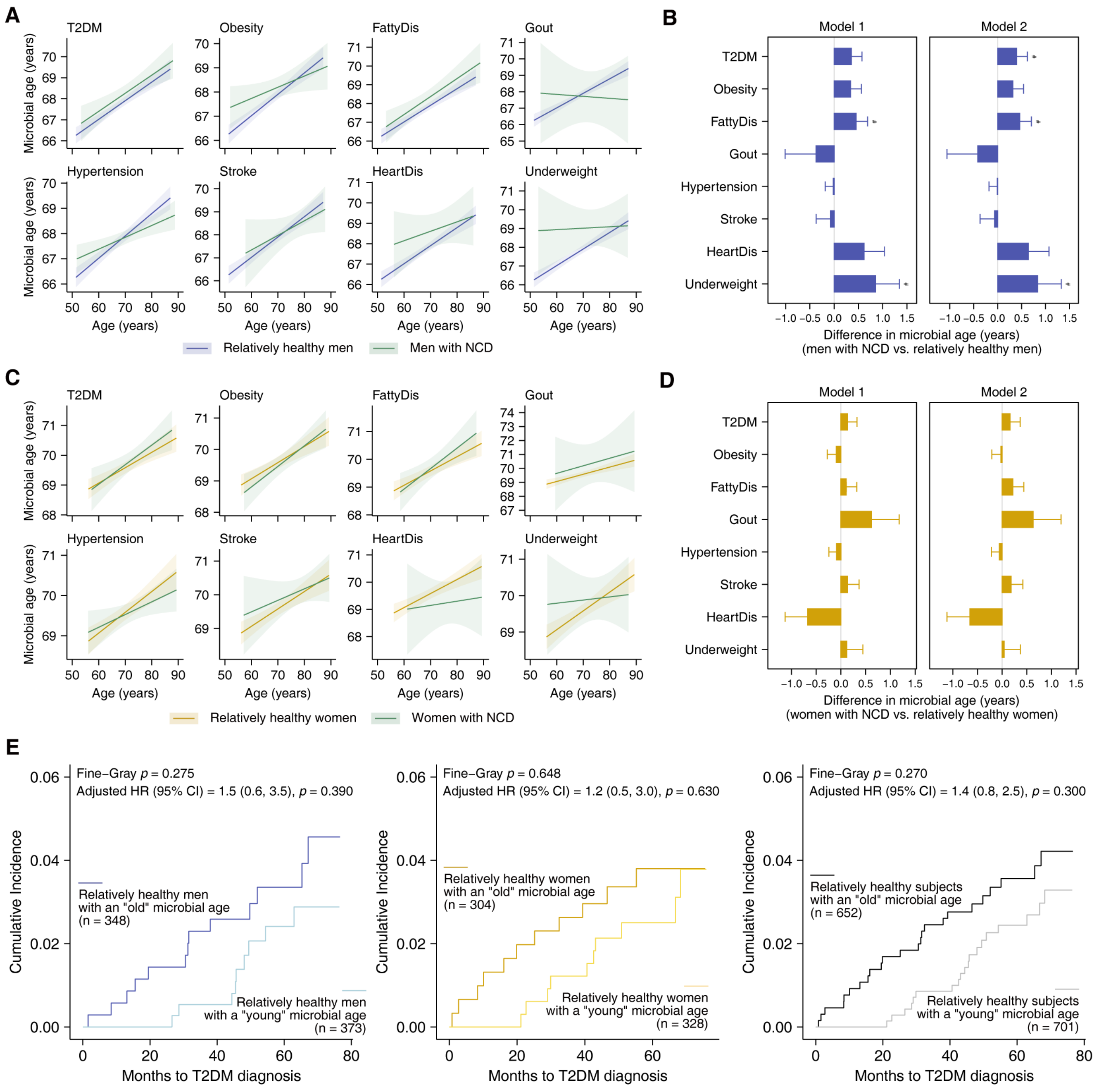

Figure S7 Microbial age estimated based on all OTUs of age-related guilds and differences by health status in men and women. (A and B) microbial age in men with or without major chronic diseases, estimated based on 266 OTUs in 24 age-related guilds in men. (C and D) microbial age in women with or without major chronic diseases, estimated based on 376 OTUs in 27 age-related guilds in women. In (A) and (C), linear fit curves of chronological age with microbial age were plotted, with 95% confidence intervals shaded. In (B) and (D), differences in microbial age and their standard errors were assessed using multivariate linear models adjusting for age (Model 1), and additionally for body mass index, total energy intake, education level, regular exercise, smoking, and regular alcohol drinking (Model 2). (E) Cumulative incidence curves of type 2 diabetes in relatively healthy subjects with an "old" or a "young" microbial age. The difference in cumulative incidence was tested using the Fine-Gary model. Hazard ratios (HRs) were estimated using a Cox proportional hazard regression model considering the competing risk of all-cause death and adjusted for age, sex (only for all subjects), body mass index, total energy intake, education level, regular exercise, smoking, and regular alcohol drinking.

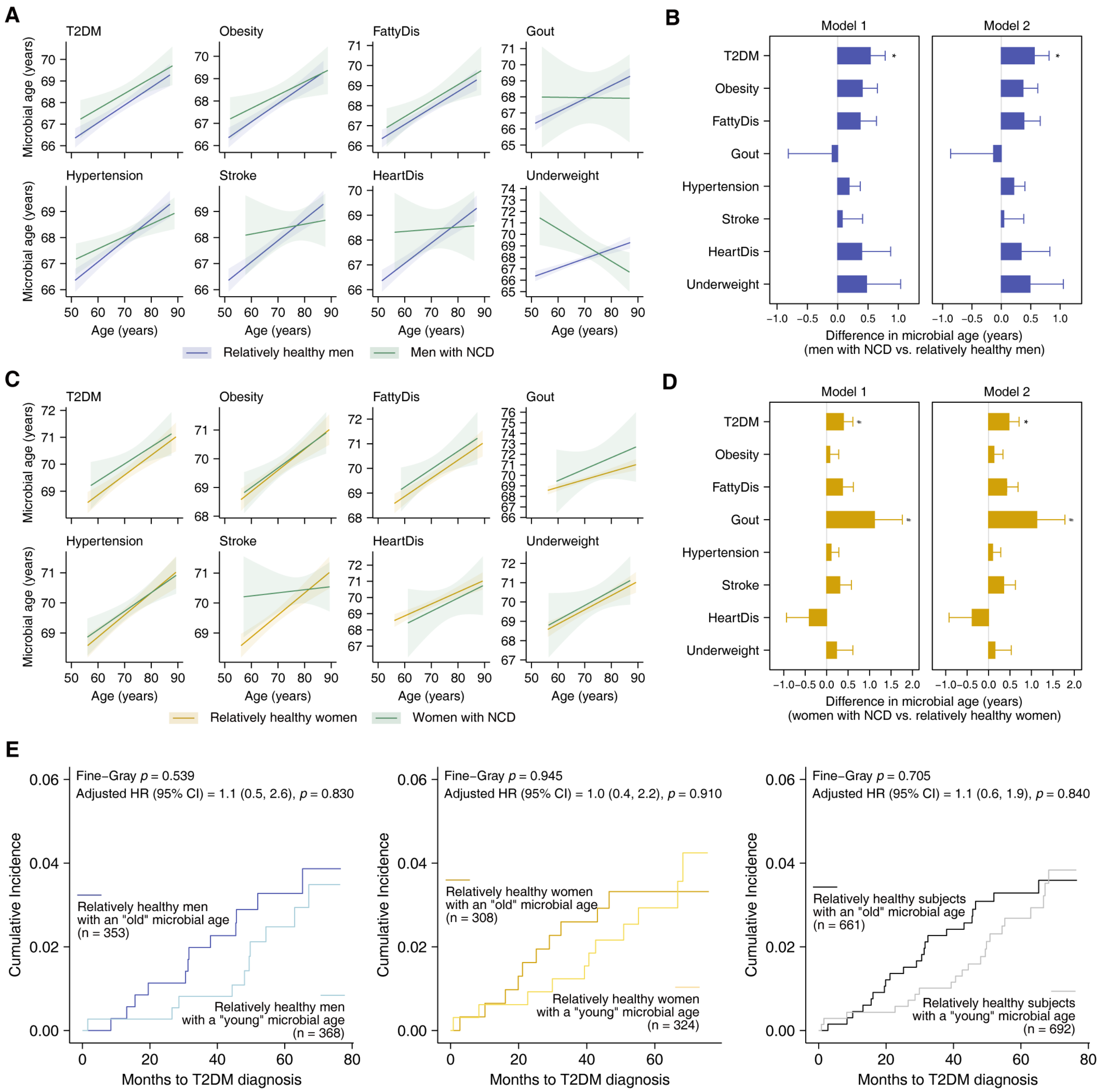

Figure S8 Microbial age estimated based on significant genera and differences by health status in men and women. (A and B) microbial age in men with or without major chronic diseases, estimated based on 27 age-related genera in men. (C and D) microbial age in women with or without major chronic diseases, estimated based on 25 age-related genera in women. In (A) and (C), linear fit curves of chronological age with microbial age were plotted, with 95% confidence intervals shaded. In (B) and (D), differences in microbial age and their standard errors were assessed using multivariate linear models adjusting for age (Model 1), and additionally for body mass index, total energy intake, education level, regular exercise, smoking, and regular alcohol drinking (Model 2). (E) Cumulative incidence curves of type 2 diabetes in relatively healthy subjects with an "old" or a "young" microbial age. The difference in cumulative incidence was tested using the Fine-Gary model. Hazard ratios (HRs) were estimated using a Cox proportional hazard regression model considering the competing risk of all-cause death and adjusted for age, sex (only for all subjects), body mass index, total energy intake, education level, regular exercise, smoking, and regular alcohol drinking.
